# “Alba” Dentists Convicted

**Published:** 1903-08

**Authors:** 


### "ALBA" DENTISTS CONVICTED.

On July 2 Judge Beitler, of Philadelphia, sentenced George C. Courtright, president of the "Alba Dental Company," and William Powell, its manager, to one year's and three months' imprisonment, respectively, upon their conviction of conspiracy to defraud.

Louis Solomon, a dental student employed by the concern, who had been convicted of practising dentistry without a license and without being registered, was at first fined fifty dollars and costs, but was later discharged, sentence being suspended.

The conviction was primarily due to the charge of a woman patient that her jaw had been fractured in the attempt to extract a supposed root. It was claimed by the prosecution that the supposed root was a portion of the bone, the fracture of which caused a long course of surgical treatment.

The committee having the matter in charge are to be congratulated in having secured these convictions. It has shown that the law can be enforced in Pennsylvania, and now that the beginning has been made, what is to prevent others equally guilty being brought to the bar of justice?

